# Caregivers’ perspectives and experiences of withdrawing acetylcholinesterase inhibitors and memantine in advanced dementia: a qualitative analysis of an online discussion forum

**DOI:** 10.1186/s12904-018-0387-0

**Published:** 2019-01-17

**Authors:** Carole Parsons, Sarah Gamble

**Affiliations:** 1 School of Pharmacy, Queen’s University Belfast, 97 Lisburn Road, Belfast, BT9 7BL UK; 2 Present Address: Clear Pharmacy, Block D, 17 Heron Road, Belfast, BT3 9LE Northern Ireland

**Keywords:** Dementia, Medication, Withdrawal, Carer(s), Online discussion forum

## Abstract

**Background:**

There is considerable uncertainty surrounding the medications used to delay the progression of dementia, especially their long-term efficacy and when to withdraw treatment with these agents. Current research regarding the optimal use of antidementia medication is limited, contributing to variability in practice guidelines and in clinicians’ prescribing practices. Little is currently known about the experiences encountered by caregivers of people with dementia after antidementia medication is withdrawn.

**Aim:**

To investigate the experiences and perspectives of carers and family members when antidementia medications (cholinesterase inhibitors and/or memantine) are stopped, by analysing archived threads and posts of an online discussion forum for people affected by dementia.

**Methods:**

Archived discussions from Talking Point, an online discussion forum hosted by the Alzheimer’s Society UK, were searched for threads discussing antidementia medication withdrawal and relevant threads were analysed thematically using the Framework method. Participant demographics were not established due to usernames which ensured anonymity.

**Results:**

Four key themes emerged: (1) expectations about withdrawal, (2) method of withdrawal, (3) clinical condition on withdrawal, and (4) the effect of withdrawal on caregivers.

**Conclusions:**

Online discussion forums such as Talking Point provide dementia carers with an outlet to seek help, offer advice and share experiences with other members. The study findings highlight the complexity surrounding optimising dementia pharmacotherapy and antidementia medication withdrawal, highlighting the need for treatment to be person-centred and highly individualised.

## Background

Dementia is a progressive, chronic, neurodegenerative condition characterised by widespread neuronal cell death which results in multiple cognitive deficits across a range of domains including memory, behaviour, language, movement and executive function, and ability to recognise familiar people and objects [1, 2]. Dementia is an increasingly challenging global public health concern; it has been estimated that 46.8 million people were living with dementia in 2015 and that this will rise to 74.7 million in 2030 and 131.5 million by 2050, due to changing demographics and increasing life expectancy [3].

Current medications approved for dementia treatment alleviate associated symptoms and delay disease progression but do not provide a cure [4, 5]. The cholinesterase inhibitors (ChEIs), donepezil, rivastigmine and galantamine, are the pharmacological agents of choice for the treatment of mild to moderate Alzheimer’s disease [6], and rivastigmine is licensed for Parkinson’s Disease Dementia (PDD) [7]. These agents are also frequently prescribed off-label for use in other types of dementia, for example, vascular dementia [5]. Loss of cholinergic neurons is apparent in the pathophysiology of Alzheimer’s disease and ChEIs exert their therapeutic effect by inhibiting acetylcholinesterase at synaptic clefts [5, 8]. This increases the availability of acetylcholine to interact with postsynaptic acetylcholine receptors, enhancing cholinergic transmission [5]. Memantine is a non-competitive N-methyl-D-aspartate (NMDA) receptor antagonist licensed for use in moderate to severe Alzheimer’s disease [5, 6]. Its mechanism of action is currently unknown, but it is thought to regulate glutamate activity, preventing overstimulation of glutamate receptors whilst not affecting glutamate transmission needed for normal physiological function [5, 8].

As dementia progresses, cognitive and physical decline can adversely impact on the ability of people with dementia to conduct basic and instrumental activities of daily living, often leaving them dependent on others for care [2, 9–11]. Caregivers of people with dementia are often presented with challenging and complex needs, higher levels of dependency and morbidity in the more severe stages of the condition [2, 12, 13]. People with advanced dementia often cannot participate in decision-making about their care; consequently decisions often have to be made by their caregivers (family members, friends and next of kin) [14–20]. Making decisions on behalf of a person with dementia can be difficult and complex [20], and a source of burden and stress for these carers, who are at increased risk of developing mental health disorders, particularly depression and anxiety [21, 22]. Recent work has shown that caregivers may find the decision-making process around medication withdrawal extremely difficult [23]. This reflects the current uncertainty in the literature regarding the long-term efficacy of antidementia drugs and when to stop treatment due to the lack of high-quality randomised controlled trials of ChEI and memantine discontinuation, and the consequent variability in clinician decision-making and prescribing practices with respect to antidementia drugs [8, 24–28].

This study aimed to investigate the experiences and perspectives of carers and family members when antidementia medications (ChEIs and/or memantine) are stopped, by analysing archived discussions of Talking Point, a UK-based online discussion forum where anyone affected by dementia, including patients, carers, family members or friends, can receive support. This manuscript presents significant further analysis and discussion of preliminary findings presented previously [29].

## Methods

### Setting

Talking Point is a fully public online discussion site hosted by Alzheimer’s Society UK. It contains various categories (broad subject areas), which themselves contain forums (more specific subject areas for discussion on different topics to assist members in finding, for example, people who are in a similar situation or at a similar stage of dementia). These forums in turn contain threads. A thread is a collection of posts defined by a title containing an opening or original post which opens the dialogue of discussion. It can contain any number of posts, including multiple posts from the same members, even if they are one after the other. A post is a message enclosed into a block containing the user’s details and the date and time it was submitted. Members are permitted to edit or delete their own posts. At the time of data collection (14th April 2017), Talking Point statistics showed that there were 94,283 threads, 1,382,996 posts, 54,531 members and 844 active members.

Only individuals who have registered as members are able to post new threads or edit posts, reply to other members’ threads, edit posts, receive email notification of replies to posts and threads, and send private messages to other members. However, as a fully public online discussion site, threads and posts on Talking Point are visible to “non-member” visitors to the forum. Archived discussions are also accessible to non-member visitors without any requirement to register or “log on” to the website.

The researcher (SG) searched the forum and collected the data as a guest; member registration was not undertaken. This ensured that the degree of intrusiveness of the research was minimal; the researcher was not actively involved in online discussions either as a declared researcher or a covert participant. Additional personal data from Talking Point members were not solicited for the purposes of this research. Informed consent was not sought from the authors of the sampled posts; the Economic and Social Research Council (ESRC) Framework for Research Ethics [30] considers forums or spaces on the internet and web “that are intentionally public” may be considered “in the public domain”.

This study is reported according to the Standards for Reporting Qualitative Research (SRQR) recommendations [31].

### Procedure

Talking Point provides a standard search feature as well as an advanced search option which enables users to define specific information required to refine results returned. For the purposes of this study, threads dating from the inception of the Talking Point forum in 2005 through to February 2017 were searched by inserting keywords into the advanced search facility. Search terms were discussed and agreed by the authors (CP and SG). The following terms, and combinations thereof, were used: ‘cholinesterase inhibitor(s)’, ‘acetylcholinesterase inhibitor(s)’, ‘donepezil’, ‘rivastigmine’, ‘galantamine’, ‘memantine’, ‘NMDA antagonist’, ‘withdraw*’, ‘discontinu*’, ‘remov*’ ‘cessat*’, ‘stop*’, ‘held’, ‘deprescribing’, ‘drug holiday’, ‘advanced dementia’, ‘dementia’, ‘drug withdrawal’, ‘medication withdrawal’, ‘drug guidance’. Relevant threads and posts were copied into a Microsoft Word document and analysed thematically using the Framework method [32]. This analytical method sits within a broad family of methods often termed thematic analysis or qualitative content analysis, and is becoming an increasingly popular analytical method in healthcare research [33].

The defining feature of the Framework Method is the matrix output of rows (cases), columns (codes) and ‘cells’ of summarised data, providing a structure whereby the researcher can systematically reduce the data in order to analyse it by case and code [33]. While in-depth analyses of key themes can take place across the entire data set, the views of each respondent remain connected to other aspects of their account within the matrix so that the context of the individual’s views is retained. Comparing and contrasting data across cases as well as within individual cases is a key feature of the Framework method [34]. The researcher is able to explore data in depth while simultaneously maintaining an effective and transparent audit trail, thus enhancing the rigour of the analytical process and the credibility of the findings [35]. Framework analysis is often overseen by an experienced qualitative researcher, but inexperienced researchers and those new to qualitative research may contribute to the analysis [34].

In this study, the five-stage method of analysis developed by Ritchie and Spencer [32] was adopted; the researcher (SG; a female undergraduate pharmacy student) re-read each transcript several times to ensure familiarisation with the data (Stage 1). At this stage, irrelevant data from the initial extraction were discarded. Data were annotated with descriptive codes; for example, clinical condition on medication withdrawal, effect on withdrawal on carers. In Stage 2, core themes were identified from the descriptive codes, reviewed and refined on discussion with the first author (CP) to produce the final coding framework. The transcripts were then read again and potentially relevant text highlighted and coded in the margins (Stage 3). In Stage 4, highlighted text from posts were electronically copied and pasted into a Microsoft Word file according to the core themes. During this process, patterns within themes emerged, resulting in the production of sub-themes (for example, improvement in clinical condition on medication withdrawal, decline in clinical condition on withdrawal, complex or uncertain change in clinical condition on withdrawal). In Stage 5, findings were synthesised by combining patterns of data, mapping out connections and developing an overall structure. These interpretations were supported by illustrative verbatim quotations from the data set. Validation of analysis was performed by SG and CP (a female academic pharmacist with experience in qualitative research).

Although Talking Point recommends that members use nicknames or create usernames which ensure anonymity, all usernames were changed using a computer-generated program to produce a random list of first names, thereby providing further assurance of anonymity in presenting the study findings using illustrative verbatim quotations.

#### Ethics and governance

Ethical approval was granted by the Research Ethics Committee of the School of Pharmacy, Queen’s University Belfast (Ref: 012PMY2017). Permission to search and analyse the threads and posts from the Talking Point forum was granted by the web editors of the forum, Alzheimer’s Society, UK.

## Results

The search of the forum yielded 95 threads comprising posts from 112 users which spanned the full date range of forum activity and contained relevant information regarding the withdrawal of antidementia medication. Thematic analysis revealed four key themes: (1) the opinion of others, (2) method of withdrawal, (3) clinical condition on withdrawal and (4) the effect of withdrawal on caregivers.

### Expectations about withdrawal

Talking Point users who expressed opinions regarding the withdrawal of antidementia medications had not always experienced these situations first hand, but often based their expectations on advice from others. Forum participants were keen to impart knowledge they had gained from these individuals including healthcare professionals, family, friends or other Talking Point members. The most common vicarious experience voiced within this theme was a rapid decline following medication withdrawal:

*“…the consultant told me that once the mementine [sic] stopped working it would be like falling of [sic] a cliff regarding his symptoms and there was nothing then that would help........they were right.” (Anne).*

Others suggested that a gradual decline post withdrawal may be expected:

*“Stopped Memantine... and [healthcare professional] said to expect a gradual peaceful withdrawal from the world that could take months.” (Frederick).*

In addition, some members reported that they were advised that an irreversible decline may occur when antidementia medication was removed:

*“He told us not to take her off because she would drop like a rock and once they go down, they stay down and he can’t get them back up.” (Vicky).*

### Method of withdrawal

The way in which these drugs were withdrawn produced concern amongst carers. Members described situations where they felt the manner of drug withdrawal affected the patient’s condition, rather than the actual withdrawal itself. Two dominant methods became apparent throughout the forum; weaning and immediate withdrawal. Many members expressed distress at the perceived consequences of rapid cessation, which included deterioration in patient condition and distress:

*“I am devastated that my dad’s medication (galantamine) was stopped suddenly by a new consultant when he started to deteriorate, in favour of memantine. I think the sudden change almost certainly added to his deterioration and his distress.” (Alice).*

Others reported increased aggression upon medication withdrawal:

*“He has never before hurt anyone and we feel it was not his fault, as it was due to drug withdrawal. The consultant was very angry as he said these drugs should never be stopped suddenly.” (Agnes).*

In addition to emergence of neuropsychiatric symptoms, physical withdrawal symptoms were also observed:

*“…did stop the drug this week and Dad had severe withdrawal symptoms including shaking, convulsing, jerking, twitching, hallucinating, and was unable to sleep for 18 hours.. . I am horrified he was put through this and cannot understand why it was withdrawn totally.... instead of him being slowly weaned off it”. (Kate).*

Conversely, a small number of members reported positive experiences with immediate withdrawal of antidementia medications.

*..they told me to stop giving it immediately. Physically he is so much better and not so tired and breathless. (Evelyn).*

*... I stopped all his meds and he recovered in a matter of days…(Alison).*

### Clinical condition on withdrawal

Within this theme, carers reported that medication withdrawal impacted on the clinical condition of the patient in a range of different ways, as detailed below.

#### No clinical change on withdrawal

Many carers reported no difference in patient condition following antidementia medication cessation in comparison to during treatment:

*“We stopped MIL [mother-in-law]’s donepezil and found no difference.” (Angela).*

*“…to be honest I don’t think that stopping the Ebixa [memantine] has had any noticeable effect.” (Linda)*

#### Improvement in clinical condition on withdrawal

Many carers witnessed an improvement in patients’ clinical condition following drug cessation. These improvements ranged from amelioration in behavioural and psychological symptoms of dementia (BPSD), physical condition and functional status, to elimination of antidementia medication side-effects, or an overall impression of improved condition. Improvements in BPSD were observed across domains including mood, aggression, anxiety, agitation and communication:

*“She has much improved since then. She can talk now and makes sense most of the time.…… I feel I have got my wife back.” (Michael).*

Carers did not always describe the side-effects from antidementia medications experienced by patients, however they did discuss elimination of these side-effects when withdrawal occurred:

*“…very bad side effects…he came off them and the side effects disappeared.” (Loretta).*

Improvements in drinking, eating and overall movement were also observed by some carers:

*“All her meds were withdrawn and she was expected to slowly withdraw and pass away. Mum defied all the expectations, started eating and drinking a little more and gradually improved. She is now stable, albeit at end stages… For her I think the withdrawal of the meds has had a remarkable effect..” (Olivia).*

Carers did not always specify which aspects of a patient’s condition had improved or in some cases described a general overall improvement:

*“…but the best day of my life was when she stopped taking it... We’re now able to lead a normal life again. I think she’s better of [sic] without it.” (Robin).*

#### Decline in clinical condition on withdrawal

Many carers reported a worsening in the condition of the patient following antidementia drug removal. Deterioration was seen in behavioural and psychological symptoms, physical condition and functional status, or a general worsening in condition. Deterioriation in behavioural and psychological symptoms observed included confusion, a worsening of communication, low mood, and worsened aggression, agitation and anxiety:

*“My mum was taken off Aricept [donepezil] and deteriorated rapidly…her talking ability started to deteriorate, she lost a lot of her cognitive skills. Basically went downhill fast….” (Christine).*

Deterioration was reported in two cases following switching from a branded formulation of donepezil to a generic version or from one generic version to another:

*“He was given a different brand of the donepezil once and I noticed a deterioration in his behaviour.” (Janet).*

*“..now on a generic Donepezil….. decline in a matter of a few weeks…. and more aggressive”. (Mary).*

As many carers were uncertain about drug efficacy, some chose to use drug holidays (the intentional interruption of pharmacological treatment for a defined period with a specific clinical purpose [36]) to test medication efficacy and withdrawal effects on individuals affected by dementia:

*“…we used that period as a [sic] opportunity to see how a ‘drug holiday’ worked for my wife and, because of the deterioration I saw, our consultant was happy to continue prescribing Aricept on the grounds that ceasing the drug would have a major effect on our daily living.” (Derek).*

Carers did not always specify what aspects of patient condition declined after withdrawal. Some carers, like Tina, observed an overall decline:

*“... the results have been more scary than I could have imagined: my husband is almost unable to understand what is being said to him, or follow instructions, he is mostly unable to communicate, he is completely unable to take care of himself, struggles climbing stairs, unable to get into a car (therefore now housebound!), almost completely incontinent…” (Tina).*

These perceived negative consequences of antidementia drug withdrawal were distressing for both patients and carers, and in some cases, this resulted in the drug being restarted or, in the case of ChEIs, in memantine being prescribed.

*“it became immediately clear that the medication had been giving her a quality of life that was valuable. Without it, her cognitive functioning and her ability to communicate was even worse, which was even more frustrating for her. We restarted it within a fortnight and she returned to her previous level.” (Rebecca).*

*“Aricept [donepezil] was withdrawn… Within a week we witnessed the most incredible decline…It was a nightmare, and I felt I needed to do something…We started on a very low dose of Ebixa [memantine]” (Alistair).*

#### Complex or uncertain change in clinical condition on withdrawal

Analysis revealed that antidementia medication withdrawal affected individuals differently. Numerous carers stated that after withdrawal, one aspect of the patient’s clinical condition improved, whilst another declined:

*“She has nosedived, dementia wise, since stopping them but is happier & feeling better & easier for me to manage.” (Rod).*

*“…they were stopped. She now is more dopey and has lost some of her verbal skills but seems more aware of what is happening and is loads calmer. It makes me wonder if the drugs were a good thing.” (Fred).*

Some carers were reluctant to apportion blame to medication withdrawal for the change in patient condition, and suggested that there were other factors that could have attributed to the deterioration in condition or to development of symptoms:

*“To me he is worse, always worse. In the past month he has declined, but I cannot say if this is due to him not taking his medication, or the Exelon [rivastigmine] being stopped, or him going into hospital, or having new medication to stop his aggressiveness.” (Hannah).*

In many instances, carers expressed confusion and indecision as to whether drug withdrawal caused patient decline or whether it was the natural course of disease progression:

*“Within a couple of weeks mum had deteriorated dreadfully but of course I have no way of telling whether that was as a result of the withdrawal of the medication or just coincidence.” (Caroline).*

### Effect of withdrawal on caregivers

Making decisions about loved ones’ medications, including withdrawal, was highly emotive and a source of worry and stress for many carers, who expressed fear for what the future held:

*“I fear we are on the slippery slope to nowhere, and I am dreading the future more than ever before.” (John).*

*“ I was really worried that I was being asked to oversee all these changes. With no medical training, I was made responsible for the gradual withdrawal or introduction of a large number of pretty heavy drugs, and I was really worried that I might make a mistake or miss some potentially dangerous side effect” (Joan).*

Carers expressed feelings of guilt, uncertainty over whether they were making the right decision, and lack of support from healthcare professionals:

*“This makes me feel awful. That we shouldn’t have asked that the medication be stopped”. (Beryl).*

*“‘Fobbed off’ is exactly how I felt when the consultant phoned me with his cold and clinical decision to take my husband off Aricept” (Barbara).*

Conversely, some carers described a sense of comfort or relief that they had made the decision to stop antidementia medication:

*“she was just like her old self, calm, no confusion, no anxiety and I thought a little miracle had happened.” (Sam).*

*“I would like to say that I am happier with the situation but really I am not because she is just so so sad. I am glad however they have removed the drug….” (Richard).*

## Discussion

Online discussion forums are increasingly being recognised as an important source of support and information available to hard-to-reach groups such as carers of people with dementia who do not access support services or as a complement to other support services offered [37]. They have been employed as a source of “naturally occurring” data in a number of clinical areas [38–45] including dementia and palliative care [37, 46, 47]. To the best of the authors’ knowledge, this is the first study to analyse the content of an online discussion forum for people affected by dementia, to provide insight into the concerns and views of caregivers specifically regarding withdrawal of antidementia medications.

Thematic analysis of the threads and posts on the subject of antidementia medication withdrawal highlighted this as a highly emotive and widely deliberated issue for caregivers, with posts spanning more than a decade, thus illustrating that it has been and continues to be a source of concern.

The evidence base to guide discontinuation of antidementia medications is limited; no blinded RCTs of discontinuation versus continuation of memantine have been conducted to date [28]. Systematic reviews and meta-analyses of ChEI discontinuation [27, 28] suggest a clinically significant worsening in cognitive symptoms after ChEI discontinuation, and a possible worsening in neuropsychiatric symptoms. Although the quality of evidence has been assessed as low due to risk of bias, lack of generalisability and indirectness of the included studies [28], this is reflected in the current study in the caregivers’ reports of healthcare professionals’, family members’, friends’ or other Talking Point forum members’ previous experiences of antidementia medication withdrawal.

The manner in which antidementia medications were withdrawn was highlighted as a source of concern for caregivers in the current study. There is evidence to suggest that a gradual downwards titration in dose may be clinically appropriate [8, 24, 27, 48, 49], with reports of adverse effects or discontinuation syndrome if medications are withdrawn abruptly [28, 50–55]. This is reflected in the caregivers’ descriptions of negative consequences for patients when antidementia medications were suddenly withdrawn.

Due to limited numbers and quality of trials to guide treatment recommendations, there is considerable clinical uncertainty regarding use of antidementia medications in the more severe stages of dementia, and how and when they should be discontinued [8, 24–28, 56–58]. Further, these trials often did not include people with advanced/end-stage dementia [28]. This clinical uncertainty is reinforced by the range of differing experiences of medication withdrawal reported by the caregivers in the current study. Some reported no observable difference in patient condition after antidementia medication cessation. Others, though notably fewer in number, reported an improvement in terms of elimination of side-effects associated with the antidementia medication or improved communication, eating and drinking or movement. The majority of caregivers, however, described a decline in the clinical condition of the patient, in relation to BPSD, physical condition, functional status or an overall general decline. In some cases, the extent and consequences of decline associated with antidementia medication withdrawal resulted in reinstatement of previous ChEI treatment or, if physicians felt the dementia had progressed, prescription of memantine. This is reflected in the literature in the area and in clinical and treatment guidelines [28, 59]. The difficulty in quantifying the ongoing benefit of antidementia medications in people with dementia is well recognised and it has been suggested that a trial withdrawal is useful in identifying patients who are still benefiting from the medication [60].

In this study, caregivers reported that attempting a drug holiday resulted in decline in the clinical condition of the patient, a finding similar to the open-label, industry-sponsored study by Doody et al., in which temporary drug cessation was reported to have a detrimental effect on cognition that was not fully recovered when the medication was re-initiated [61]. Caregivers also reported clinical decline if medication was switched from one branded drug to another or to a generic counterpart. Previous work has highlighted the possibility of clinical deterioration for people with mild-to-moderate dementia when switching ChEIs [62], but did not consider the advanced/end of life stages. However, the study finding of a difference on switching from a branded ChEI to a generic version has not been reported in the literature, and contrasts with work reporting no significant difference between generic and branded donepezil on quality of life of patients with Alzheimer’s disease [63]. This finding suggests further exploration of the effects of switching between generic and branded antidementia drugs on the clinical condition of patients in the advanced stages of dementia is warranted.

Improvement in some aspects of the patient condition accompanied by deterioration in other areas (a complex change in the patient’s clinical condition) was described by a number of caregivers in the current study. Current clinical guidelines do not allow for a complex change in patient condition, but do recognise that it can be difficult to determine whether there is ongoing benefit from a medication due to the progressive and fluctuating nature of dementia [28, 64, 65]. Previous work suggested that caregivers and proxy decision-makers of people with dementia are uncertain about benefits of antidementia medications as there is no way to measure their effects objectively, but these medications provided a sense of hope [23, 66–71]. Carers in this study echoed this, reporting that they were often unsure if decline was a result of antidementia drug withdrawal or if it was part of the natural progression of the disease.

The final key theme emerging in this study was the burden, worry, stress and guilt caregivers experienced when making decisions about loved ones’ medications. This reflects the literature in the area of decision-making on behalf of people with dementia; making these decisions is recognised to be a complex and difficult process [20], and the emotional burden involved in the responsibility of making decisions about medications has been acknowledged [66, 72–76], as has the guilt and self-remonstration experienced by those who feel that their decision has had a detrimental impact on their loved one [72].

### Strengths of the study

The Talking Point online discussion forum provided a large data source for this study. The data collected and analysed demonstrated a wide range of different types of interactions between Talking Point members, ranging from sympathetic and supportive to dissenting and challenging. User anonymity allowed members to be open in sharing their feelings and experiences, minimising the risk of social desirability bias.

### Limitations of the study

Data were collected from one online discussion forum hosted by the Alzheimer’s Society UK. These were therefore restricted to carers who had internet access, were aware of the facilities offered by Talking Point and could communicate in the English language. Demographics of carers who posted on the website were not available, and assumptions cannot be made on generalisability or transferability of the results to the wider population. Further, due to the nature of data collection, it was not possible to clarify meanings or obtain further explanation of posts. Carers’ interpretation of effects of withdrawal were individualised and subjective; for example, what one carer described as a major deterioration in patient condition may not have been ascribed the same severity by another. In addition, any threads which did not contain correct drug spelling on at least one occasion, or which solely used brand names rather than generic nomenclature for the ChEIs and memantine, were omitted by the search thus potentially omitting relevant data. However, posts including brand names and misspelt drug names were identified through searching using the other keywords referring to stopping, withdrawing or withholding medications, which provides some reassurance that missing data are limited.

## Conclusion

This study is the first to explore caregivers’ views and experiences of withdrawing antidementia medication in people with advanced/end-stage dementia by analysing the content of an online discussion forum for people affected by dementia. It highlights the widely varying response to treatment with antidementia medication and to subsequent medication withdrawal experienced by people with dementia, confirming the importance of a highly individualised and person-centred approach in clinicians’ and caregivers’ decisions regarding the withdrawal of antidementia medications.

## Abbreviations

BPSD: Behavioural and psychological symptoms of dementia
ChEI: Cholinesterase inhibitor
